# *Schisandra chinensis* and* Morus alba* Synergistically Inhibit In Vivo Thrombus Formation and Platelet Aggregation by Impairing the Glycoprotein VI Pathway

**DOI:** 10.1155/2017/7839658

**Published:** 2017-01-18

**Authors:** Dong-Seon Kim, Muhammad Irfan, Yoon-Young Sung, Seung Hyung Kim, Sun Haeng Park, Young Hyun Choi, Man Hee Rhee, Ho Kyoung Kim

**Affiliations:** ^1^Korea Institute of Oriental Medicine, Daejeon 34054, Republic of Korea; ^2^Laboratory of Veterinary Physiology and Cell Signaling, College of Veterinary Medicine, Kyungpook National University, Daegu 41566, Republic of Korea; ^3^Institute of Traditional Medicine & Bioscience, Daejeon University, Daejeon 34520, Republic of Korea; ^4^Department of Biochemistry, Dongeui University College of Korean Medicine, Busan 614-052, Republic of Korea

## Abstract

*Morus alba *L. (MAL) extract has been used in traditional medicine for its cardioprotective and antiplatelet effects, while another herbal remedy,* Schisandra chinensis* (SCC), has been reported to have anti-inflammatory and antioxidant properties. We evaluated underlying cellular changes exerted by extracts of these plants on platelet function and effects of SCC + MAL on in vivo thrombus formation using AV shunt and tail thrombosis-length models in rats. In vitro platelet aggregation, granule secretion, and [Ca^2+^]_*i*_ release assays were carried out. The activation of integrin *α*_IIb_*β*_3_ and phosphorylation of downstream signaling molecules, including MAPK and Akt, were investigated using cytometry and immunoblotting, respectively. Scanning electron microscopy (SEM) was used to evaluate changes in platelet shape and HPLC analysis was carried out to identify the marker compounds in SCC + MAL mixture. In vivo thrombus weight and average length of tail thrombosis were significantly decreased by SCC + MAL. In vitro platelet aggregation, granule secretion, [Ca^2+^]_*i*_ release, and integrin *α*_IIb_*β*_3_ activation were notably inhibited. SCC + MAL markedly reduced the phosphorylation of MAPK pathway factors along with Akt. HPLC analysis identified four marker compounds: isoquercitrin, astragalin, schizandrol A, and gomisin A. The extracts exerted remarkable synergistic effects as natural antithrombotic and antiplatelet agent and a potent drug candidate for treating cardiovascular diseases.

## 1. Introduction

Platelet activation underlies thrombotic cardiovascular diseases (CVD) and events such as coronary artery disease (CAD), atherosclerosis, myocardial infarction, and thrombosis which are responsible for increased morbidity and mortality, and their prevalence in western world is increasing at a high pace [1]. Platelet activation plays a critical role in hemostasis, but inappropriate activation can lead to development of serious cardiovascular disorders, and is the causal factor in atherosclerosis [2, 3]. Rupture of atherosclerotic plaque can lead to CAD development and death [4]. Therapeutic antiplatelet agents have been shown to reduce the incidence of CVD [5]. Many antiplatelet drugs, including aspirin and clopidogrel, exert side effects such as gastric bleeding and hemorrhage, limiting their usage [6]. Therefore, there is need to develop better and safe approaches to manage thrombosis and CVD. One of the approaches may include use of natural products, such as plant extracts as antithrombotic agents and anticoagulants [7]. The development of these diseases may be slowed down through the use of dietary supplements and natural products [8]. Ethnomedicinal applications are gaining popularity in the treatment of CVD [7]. Some dietary and herbal compounds have been shown to decrease the risks associated with CVD [9].

*Morus alba* L. has been traditionally used in China, Japan, Korea, and other parts of Asia as an herbal tea and medicine, and its leaves extract has been shown to exert antiplatelet effects [10, 11].* Schisandra chinensis* is known to exhibit strong anti-inflammatory and antioxidant properties. It has also long been used in traditional Chinese and Korean herbal medicine for its tonic, antioxidant, and sedative effects [12–14]. In a previous report,* S. chinensis* showed antagonistic activity against platelet-activating factor (PAF) [15]. To date, there is no detailed evidence available on the antiplatelet activity of this compound. In this study, we demonstrated synergistic antithrombotic effects of these herbal compounds, which decrease platelet activity as well as thrombus formation by pharmacological suppression of platelet function.

## 2. Material and Methods

### 2.1. Chemicals

Collagen was purchased from Chrono-log (Havertown, PA, USA). ASA, Fura-2/AM, *κ*-carrageenan, and dimethyl sulfoxide (DMSO) were obtained from Sigma-Aldrich (St. Louis, MO, USA). ATP Assay Kit was obtained from Biomedical Research Service Center (Buffalo, NY, USA). Fibrinogen Alexa Fluor 488 conjugate was obtained from Molecular Probes (Eugene, OR, USA). Antibodies against phospho-p44/42, p44/42, phospho-p38, p38, phospho-SAPK/JNK, SAPK/JNK, phospho-Akt, and Akt were acquired from Cell Signaling Technology (Beverly, MA, USA). HPLC-grade reagents, acetonitrile, and water were obtained from J. T. Baker (Phillipsburg, NJ, USA). All chemicals were of reagent-grade.

### 2.2. Preparation of* S. chinensis* and* Morus alba* L. Extracts

SCC and MAL were purchased from Omniherb Co. (Daegu, Korea) and were authenticated by the classification and identification committee of Korea Institute of Oriental Medicine (KIOM) based on macroscopic and microscopic features. The same extraction procedure was applied for both plants. One kilogram of dried plant leaves was extracted twice at 80°C for 3 h with 70% ethanol (v/v). The extracts were filtered and evaporated in a rotary evaporator under low pressure. The SCC extract and MAL extract yields were 34% and 6.3%, respectively. Extracts were dissolved in water and DMSO for in vivo and in vitro studies, respectively.

### 2.3. Animals and Dosing

Seven-week-old male Sprague Dawley rats (*n* = 74) weighing 250–260 g were purchased from Orient Co. (Seoul, Korea). Rats were acclimatized for 1 week before conducting experiments and in an air conditioned animal room with 12 h light and 12 h dark cycle at temperature and humidity of about 22 ± 2°C and 55 ± 10%, respectively. Twenty-four rats were randomly divided into four groups (*n* = 6) for the arteriovenous (AV) shunt model: negative control,* S. chinensis* (SCC),* M. alba* L. (MAL), and SCC + MAL. Fifty rats were divided into five groups (*n* = 10) for the tail thrombosis-length model: negative control, SCC, MAL, SCC + MAL, and ASA. Rats received 200 mg/kg SCC, MAL, or SCC + MAL orally once a day for 3 days (AV shunt model groups) and 7 days (tail thrombosis-length model groups), except the negative control groups.

### 2.4. AV Shunt Model

The antithrombotic activity of SCC, MAL, and SCC + MAL extracts was assessed in a rat extracorporeal shunt model as previously described [16], with a little modification. Briefly, rats were administered with 200 mg/kg (human equivalence dose (HED)) [17] of SCC, MAL or SCC + MAL (1 : 1) extracts or vehicle orally once a day for three days. Two hours after the last administration, rats were anesthetized with urethane (1.75 g/kg i.p.). The left carotid artery and right jugular vein were exposed by incision and the two ends of extracorporeal shunt inserted into them. The shunt comprised of 12 cm polythene tubes (0.81 mm external and 0.58 mm internal diameter) joined by 5 mm silicone rubber plugs to a 6 cm polyvinyl tube with 3 mm of internal diameter. A cotton thread was held between two plugs so that it remained longitudinally oriented in the blood flow through cannula. The tube was filled with normal saline before cannulation and the shunt was left in place for 15 min after the extracorporeal circulation started. Thread was removed by stopping the blood flow to obtain thrombus that had formed, separated from thread and weighed.

### 2.5. *κ*-Carrageenan-Induced Rat Tail Thrombosis Model

To further confirm the in vivo antithrombotic activity of SCC, MAL, and SCC + MAL, a carrageenan-induced rat tail thrombosis model was used as previously described [18] with a minor modification. Rats were administered with 200 mg/kg of SCC, MAL, or SCC + MAL (1 : 1) extracts, 50 mg/kg of ASA, or vehicle, orally once a day for 7 days. On the 4th day, 30 min after oral administration, rats were injected with a single dose of *κ*-carrageenan (20 mg/kg BW i.p.) dissolved in physiological saline. Tails were observed for redness, swelling, and thrombosis. Rats were monitored for development of thrombosis and treated for a further 3 days. On the 7th day and 72 h following a *κ*-carrageenan injection, thrombosis length was recorded for each rat in all groups.

### 2.6. Platelet Preparation and Aggregation

Whole blood was obtained from SD rats and transferred to anticoagulant acid/citrate/dextrose (ACD) solution (21 mM citric acid, 85 mM trisodium citrate, and 83 mM dextrose) containing tubes. Platelet rich plasma (PRP) was obtained by centrifuging the blood at 170 ×g for 7 min and PRP was again centrifuged two times at 350 ×g to isolate washed platelets. Tyrode's buffer solution (137 mM NaCl, 12 mM NaHCO_3_, 5.5 mM glucose, 2 mM KCl, 1 mM MgCl_2_, and NaHPO_4_, pH 7.4) was added to adjust the platelet concentration at 3 × 10^8^/mL to proceed for platelet aggregation assay. All the procedure for preparation was conducted at room temperature (23 ± 2°C).

Platelet aggregation was performed as previously described method [19] and the extent of aggregation was measured by light transmission aggregometry (Chrono-log, Havertown, PA, USA). Washed platelets were preincubated for 2 min at 37°C along with 1 mM CaCl_2_ and stimulated with collagen; then solution was incubated for more 5 min with continuous stirring. The vehicle ratio was not more than 0.05%.

### 2.7. Scanning Electron Microscopy Analysis

Field Emission Scanning Electron Microscope (SU8220, Hitachi) was used to assess aggregation ultrastructure image at center for scientific instrument, Kyungpook National University, Daegu, Korea. After the platelet aggregation assay, the platelets mixtures were fixed using 0.5% paraformaldehyde (1st fixation) and osmium tetroxide (2nd fixation), dehydrated using ethanol, freeze-dried, and scanned.

### 2.8. [Ca^2+^]_*i*_ Measurement

[Ca^2+^]_*i*_ concentration was measured with Fura-2/AM as previously described [20]. Briefly, platelets were preincubated with 5 *µ*M Fura-2/AM for 1 h at 37°C and washed. Platelets were then preincubated for 1 min along with 1 mM CaCl_2_ at 37°C and stimulated with agonist for 2 min. Fluorescence was recorded using spectrofluorometer (F-2500; Hitachi, Japan) and [Ca^2+^]_*i*_ was calculated as previously described method by Schaeffer and Blaustein [21] using the following formula: [Ca^2+^]_*i*_ in cytosol = 224 nM × (*F* − *F*_min_)/(*F*_max_ − *F*), in which 224 nM is the dissociation constant of the Fura-2-Ca^2+^ complex and *F*_min_ and *F*_max_ represent the fluorescence intensity levels at very low and very high Ca^2+^ concentrations, respectively.

### 2.9. Granule Secretion

Washed platelets were preincubated along with 1 mM CaCl_2_ for 2 min at 37°C and stimulated with agonist. Supernatants were obtained by centrifuging the platelet mixture after the aggregation reaction terminated, and ATP secretion was measured in a luminometer (GloMax 20/20, Promega, Madison, WI, USA) using an ATP Assay Kit (Biomedical Research Service Center).

### 2.10. Flow Cytometry

Flow cytometry was used to quantify fibrinogen Alexa Fluor 488 conjugate binding to platelets. Washed platelets were preincubated for 2 min along with 0.1 mM CaCl_2_, stimulated with agonist for 5 min and then incubated with fibrinogen Alexa Fluor 488 (20 *µ*g/ml) for 5 min at room temperature, and then fixed with 0.5% paraformaldehyde for 30 min at 4°C. Platelets were suspended in 400 *µ*L phosphate buffered saline (PBS) after three times washing with PBS. The fluorescence was analyzed using a FACS Caliber cytometer (BD Biosciences, San Jose, CA, USA), and data were analyzed using CellQuest software (Becton Dickinson Immunocytometry Systems, San Jose, CA, USA).

### 2.11. Immunoblotting

After platelet aggregation, suspension was solubilized in sample lysis buffer (0.125 M Tris-HCl [pH 6.8], 2% SDS, 2%  *β*-mercaptoethanol, 20% glycerol, 0.02% bromophenol blue, 1 *µ*g/mL phenyl methyl sulfonyl fluoride [PMSF], 2 *µ*g/mL aprotinin, 1 *µ*g/mL leupeptin, and 1 *µ*g/mL pepstatin A) and centrifuged. Protein concentrations were measured using BCA assay (PRO-MEASURE; iNtRON Biotechnology, Seoul, Korea) and total cell proteins (35 *µ*g) were isolated from lysates using 10% SDS-PAGE and transferred to polyvinylidene difluoride membranes in transfer buffer (25 mM Tris [pH 8.3], 0.2 M glycine, and 20% methanol). Membranes were blocked in TBS-T containing 5% dry skim milk and probed with primary and secondary antibodies, and then antibody binding was visualized using enhanced chemiluminescence (Advansta, CA, USA).

### 2.12. High-Performance Liquid Chromatography (HPLC) Analysis to Identify the Marker Compounds in SCC + MAL

The mixture was analyzed by reverse phase-HPLC (Waters Alliance 2695 system, Waters Co., Milford, MA, USA) combined with a 2996-photodiode array detector. A Phenomenex Luna C18 column (250 × 4.6 mm; 5 *µ*m particle size, Phenomenex, Torrance, CA, USA) was used for the stationary phase; the mobile phase was composed of 0.1% (v/v) trifluoroacetic aqueous solution (A) and acetonitrile (B). The elution conditions were as follows: at *t* = 0 min, the mobile phase was held for 10 min and consisted of 90% A and 10% B. A gradient was applied for 10 to 60 min, to 40% A and 60% B, which was followed by a wash with 100% B for 10 min and a 15 min equilibration period at 90% A and 10% B. A flow rate of 1.0 mL/min at a temperature of 40°C, and an injection volume of 20 *µ*L were maintained throughout the analysis for the separation.

The compounds were recognized by comparing retention times and UV spectra of the peaks of HPLC/PDA chromatograms to those of commercially available standards. For each compound, peak area was determined at a wavelength of 240 nm. The calibration curve of the standards, ranging from 12.5 to 200 *µ*g/mL (5 levels) for each standard, revealed a good linearity with the linear correlation coefficient; *R*^2^ values exceeded 0.99 (peak area versus concentration). Quantitation was performed in comparison to a mixture of external standards of known concentrations that was analyzed in duplicate before and after the batch samples and the peak areas were used to calculate the concentrations of the compounds in the samples.

### 2.13. Statistical Analysis

One-way analysis of variance (ANOVA) followed by post hoc Dunnett's test was used to determine statistical significance in the observed differences (SAS Institute Inc., Cary, NC, USA). All data are presented as the mean ± standard error of the mean (SEM) and *P* value of 0.05 or less was considered statistically significant.

## 3. Results

### 3.1. In Vivo Effects on Thrombus Formation

**Figure 1:**
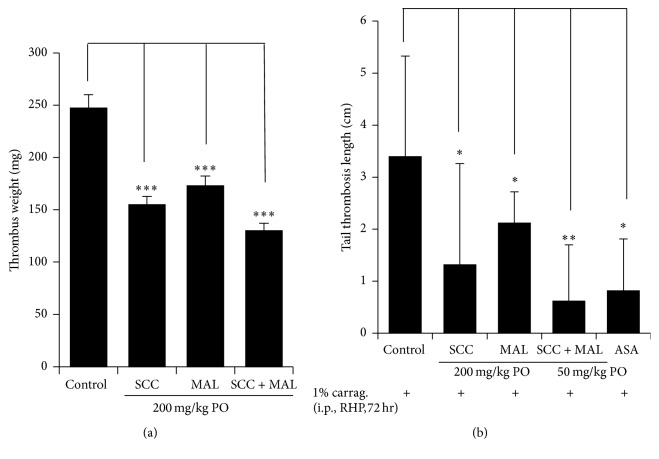
In vivo effects of the SCC + MAL mixture on thrombus formation. (a) The arteriovenous (AV) shunt model was used to evaluate in vivo antithrombotic activity. Rats received SCC, MAL, or SCC + MAL extracts or vehicle, orally once a day for 3 days. On the 3rd day, 2 h after oral administration, blood circulation in the cannula carried out for 15 min, and thrombus weight determined immediately. (b) Rat tail thrombosis-length model was used to evaluate inhibitory effects on tail thrombosis. Rats received SCC, MAL, or SCC + MAL extracts, ASA, or vehicle, orally once a day for 7 days. On the 4th day, 30 min after oral administration, rats were injected with a single dose of *κ*-carrageenan (20 mg/kg BW i.p.) dissolved in physiological saline. Bar graph shows the mean ± SEM for AV shunt (*n* = 3 rats in each group) and tail thrombosis (*n* = 10 rats in each group). ^*∗*^*P* < 0.05,  ^*∗∗*^*P* < 0.01, and ^*∗∗∗*^*P* < 0.001 versus control.

AV shunt thrombosis models have been used to evaluate in vivo antithrombotic effects [3, 16]. We therefore studied the effects of SCC, MAL, and SCC + MAL on thrombus formation in an extracorporeal shunt model. As shown in Figure 1(a), all treated groups displayed significantly reduced arteriovenous shunt thrombus formation compared to that observed for negative controls (*P* < 0.001). Moreover, the mixture showed more potent thrombus reduction (47 ± 2.3%) than SCC or MAL (37 ± 2.3 or 32 ± 0.2%, respectively) alone did (*P* < 0.001).

A *κ*-carrageenan-induced tail thrombosis-length model in rats is useful for evaluating antithrombotic effects [22]. We measured and recorded thrombus length; swelling and redness were observed in the negative control groups within 4-5 h of *κ*-carrageenan injection, but not in the treatment groups. Length of tail thrombosis was recorded 72 h after *κ*-carrageenan injection. As shown in Figure 1(b), the negative control group displayed a significant increase in thrombosis length compared to that observed in rats treated with ASA (77 ± 21%; *P* < 0.05), which was used as a reference antithrombotic agent. The SCC and MAL groups showed notable decreases in thrombosis length,* that is*, 62 ± 39% and 39 ± 11% (*P* < 0.05), respectively, compared with control, while the SCC + MAL group displayed a significant reduction in thrombosis length (83 ± 22%; *P* < 0.01) compared to that reported for all other groups, indicating that the mixture exerted synergistic inhibitory effects on *κ*-carrageenan-induced thrombus formation and had potent antithrombotic properties.

### 3.2. Effects on Agonist-Induced Platelet Aggregation

**Figure 2:**
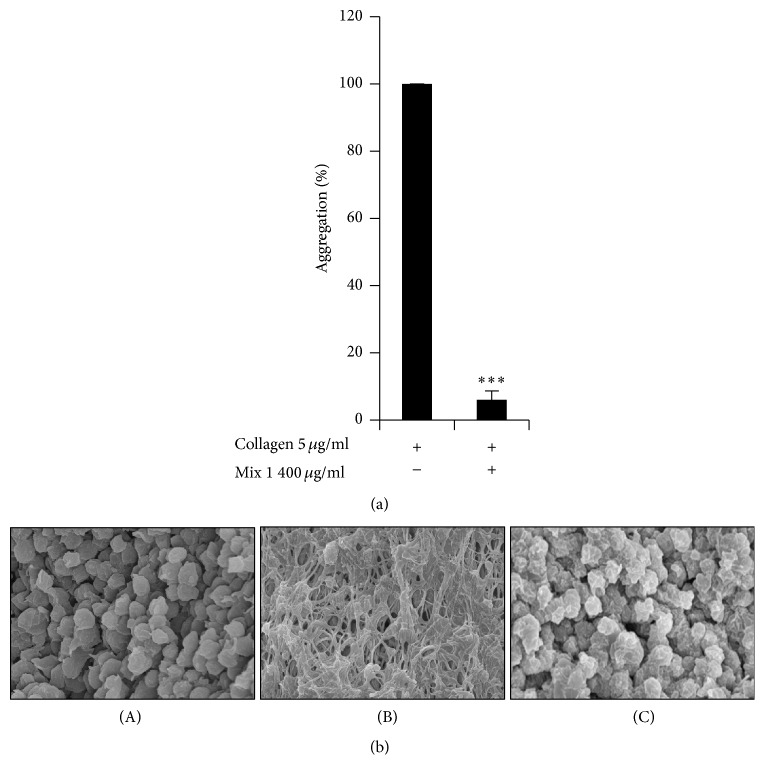
(a) SCC + MAL inhibited collagen-induced platelet aggregation. Aggregation was quantified and expressed as a percentage. Graph shows the mean ± SEM of at least four independent experiments. (b) Representative scanning electron microscopy images of resting state or collagen-treated platelets incubated with or without SCC + MAL: basal (A), 5 *µ*g/mL of collagen (B), and 400 *µ*g/mL of SCC + MAL (C). ^*∗∗∗*^*P* < 0.001 compared to the agonist control.

To further confirm the antiplatelet activity of SCC + MAL, we investigated the underlying mechanism using in vitro techniques. Light transmission aggregometry was employed to assess the effect of SCC + MAL on agonist-induced platelet aggregation. First of all we evaluated whether our compound affected various ligands (ADP, collagen, or thrombin) but SCC + MAL only inhibited collagen-induced platelet aggregation (data not shown). It is also consistent with our previous study [11], which have shown that MAL only inhibited collagen-induced platelet aggregation and did not affect other ligands. Moreover, we tried different dosage of SCC + MAL and 400 *µ*g/ml found to inhibit maximum platelet aggregation (data not shown). Therefore, we employed collagen as ligand and found that SCC + MAL dramatically inhibited collagen-induced platelet aggregation by 94 ± 03% compared to the control value (*P* < 0.001) (Figure 2).

### 3.3. Effects on [Ca^2+^]_*i*_

**Figure 3:**
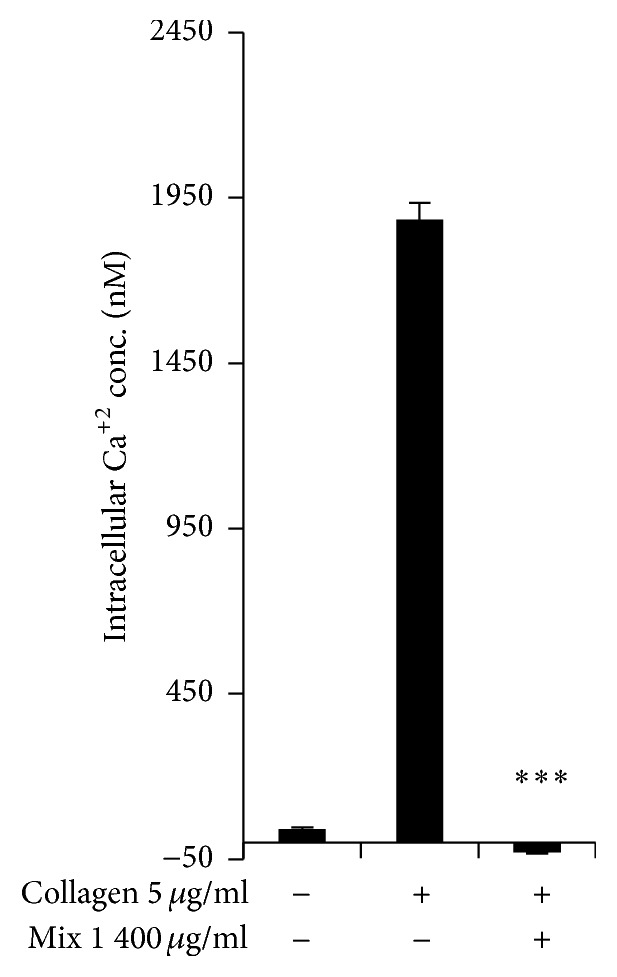
The inhibitory effect of SCC + MAL on [Ca2+]_*i*_ induced by collagen. Washed platelets (3 × 10^8^/mL) were incubated with a calcium fluorophore (5 *µ*M, Fura-2/AM) and stimulated with collagen (5 *µ*g/mL). The results are presented as the mean ± SEM of at least four independent experiments. ^*∗∗∗*^*P* < 0.001 compared to the agonist control.

[Ca^2+^]_*i*_ elevation plays a vital role in platelet aggregation and degranulation and modulates platelet functions such as thrombus formation and platelet shape change [14]. To assess whether the SCC + MAL mixture inhibited [Ca^2+^]_*i*_, an intracellular calcium mobilization assay was performed. As shown in Figure 3, SCC + MAL completely blocked [Ca^2+^]_*i*_ mobilization (*P* < 0.001).

### 3.4. Effects on Granule Secretion (ATP Release)

**Figure 4:**
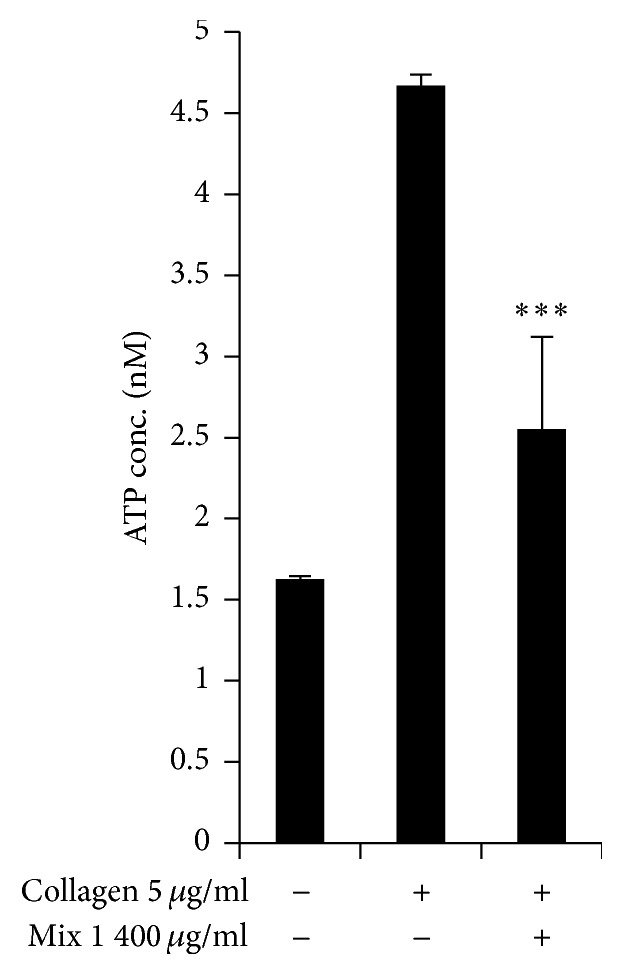
Inhibitory effects of SCC + MAL on dense granules secretions. Washed platelets (3 × 10^8^/mL) were preincubated for 2 min at 37°C in the presence of 1 mM CaCl_2_ and then stimulated with collagen. After terminating the aggregation, ATP release assay was performed. Bar graph shows the mean ± SEM of at least four independent experiments. ^*∗∗∗*^*P* < 0.001 compared to the agonist control.

Platelet activation causes the release of different granule populations such as *α*-granules and dense granules; these secretions boost platelet activation and intracellular signaling. We examined the effects of SCC + MAL on ATP release from dense granules and found that the mixture inhibited ATP release by 47 ± 15% (*P* < 0.001) (Figure 4).

### 3.5. SCC + MAL Limits Integrin *α*_IIb_*β*_3_ Mediated Inside-Out Signaling

**Figure 5:**
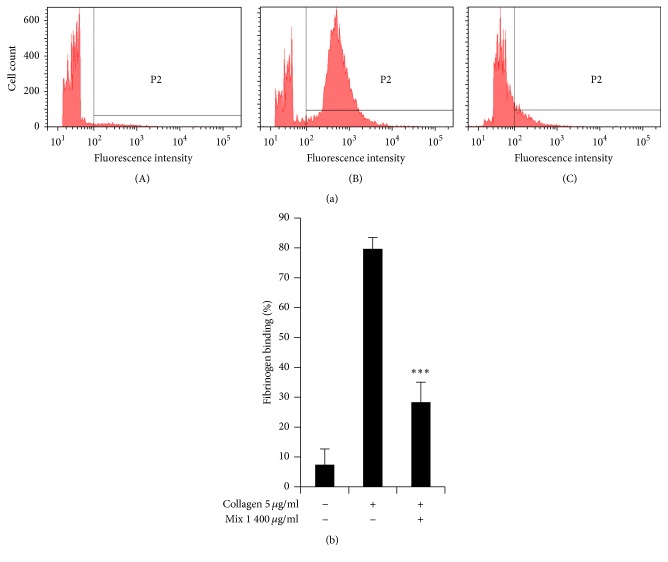
(a) The inhibitory effect of SCC + MAL on fibrinogen binding to integrin *α*_IIb_*β*_3_. Washed platelets (3 × 10^8^/mL) were preincubated for 2 min at room temperature in the presence of 0.1 mM CaCl_2_, stimulated with collagen for 5 min and fibrinogen Alexa Fluor 488 (20 *μ*g/mL), and then fixed with 0.5% paraformaldehyde at 4°C for 30 min. Representative FACS analysis results of four independent trials are shown. Basal (A), 5 *µ*g/mL of collagen (B), and 400 *µ*g/mL of SCC + MAL (C). (b) Bar graph summarizing the inhibitory effect of SCC + MAL extract on fibrinogen binding. ^*∗∗∗*^*P* < 0.001 compared to the agonist control.

Platelets express integrins, such as *α*_2_*β*_1_ (collagen receptor), *α*_IIb_*β*_3_ (fibrinogen receptor), and *α*_V_*β*_1_ (fibronectin receptor). The fibrinogen binding to integrin *α*_IIb_*β*_3_ is considered a marker of *α*_IIb_*β*_3_ inside-out signaling [23]. We investigated the effect of SCC + MAL on fibrinogen binding to integrin *α*_IIb_*β*_3_ and found that SCC + MAL notably reduced fibrinogen binding to integrin *α*_IIb_*β*_3_ by 65 ± 06% (*P* < 0.001) (Figure 5).

### 3.6. SCC + MAL Reduced MAPK and Akt Phosphorylation

**Figure 6:**
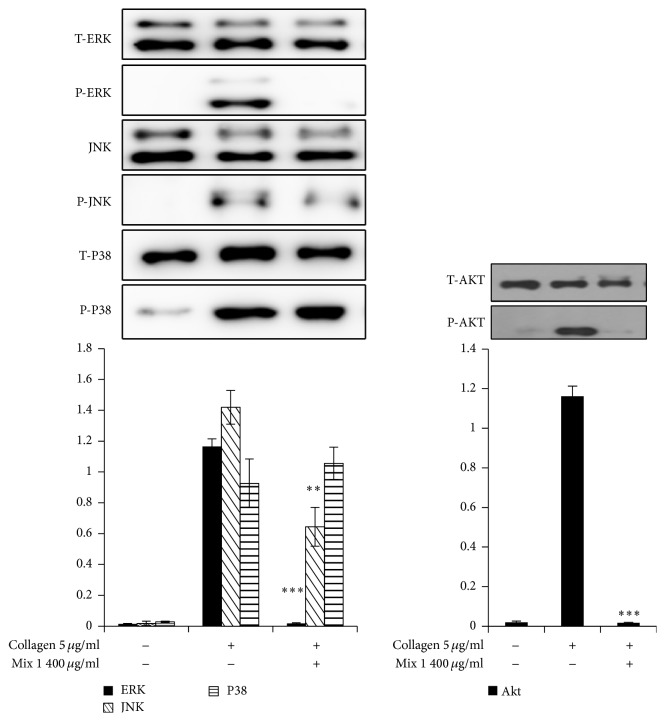
SCC + MAL reduced the phosphorylation of MAPK (i.e., ERK1/2 and JNK but not p38) and Akt. Cell proteins were extracted after aggregation was terminated, and proteins were separated using SDS-PAGE, transferred to PVDF membranes, and then probed with antibodies against total and phospho-ERK1/2, JNK, p38, and Akt. All immunoblots were carried out in at least four independent experiments. ^*∗∗*^*P* < 0.01 and ^*∗∗∗*^*P* < 0.001 compared to the agonist-treated group.

To explore the underlying intracellular signaling pathway of SCC + MAL effects on platelet activation, we evaluated its effect on protein phosphorylation. Phosphorylation of MAPKs (JNK, ERK, and P38-MAPK) is known to mediate the platelet activation pathway [24] while PI3K/Akt signaling is another important pathway in platelet activation. Therefore we investigated whether SCC + MAL decreased MAPK and PI3K/Akt phosphorylation. SCC + MAL markedly blocked collagen-induced phosphorylation of MAPKs (ERK and JNK but not P38-MAPK) and Akt, indicating that the mixture can affect the MAPK and PI3K/Akt signaling pathways (Figure 6).

### 3.7. Chromatographic Separation of SCC + MAL to Identify Marker Compounds

**Figure 7:**
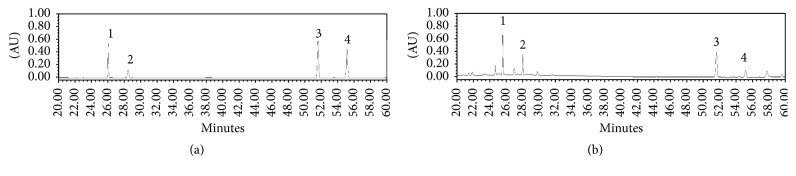
HPLC chromatograms of four standards mixture (a) at 240 nm and the 70% (v/v) ethanol extract of SCC + MAL. Isoquercitrin (1), astragalin (2), schizandrol A (3), and gomisin A (4) appeared at a retention time of approximately 25.6, 28.0, 51.6, and 55.1 min, respectively.

Marker compounds were determined from the major peaks of HPLC/PDA chromatograms in comparison with the retention times and UV spectra of commercial standards. HPLC analysis of SCC + MAL identified four compounds: isoquercitrin and astragalin were derived from* M. alba* L., and schizandrol A and gomisin A were derived from* S. chinensis*; these appeared at a retention time of approximately 25.6, 28.0, 51.6, and 55.1 min, respectively (Figure 7). SCC + MAL contained 13.6 mg/g of isoquercitrin, 5.8 mg/g of astragalin, 7.3 mg/g of schizandrol A, and 2.0 mg/g of gomisin A.

## 4. Discussion

Coronary atherosclerotic disease and other acute clinical manifestations are commonly caused by plaque disruption and subsequent thrombus formation [4]. Previous studies have suggested that prophylactic pharmacological suppression of platelet activation can inhibit the risk of prothrombotic state, slow down the development of thrombosis, and reduce the risk of CVD [5, 25]. Findings from primary prevention trials have noted that the side effects of some prophylactic regimens outweigh their benefits [26, 27]. Several studies reported that certain dietary and herbal compounds can reduce the risk factors linked to CVD [7, 9].

MAL has traditionally been used as an herbal medicine and has recently been shown to have antiplatelet and antithrombotic properties [11]. SCC has been used as a Korean herbal medicine for its antitussive, antioxidant, sedative, and tonic effects. SCC fruits and leaves contain a number of pharmacologically active compounds [28]. We therefore evaluated its antiplatelet and antithrombotic properties in combination with MAL and explored the underlying mechanism of their effects on platelet function modulation, by assessing the in vivo antithrombotic activity of both extracts individually and as a mixture.

Carrageenan, which is composed of repeating units of 3,6-anhydro-D-galactose and D-galactose, is a straight-chain, sulfur-containing macromolecular polysaccharide. *κ*-carrageenan is broadly used for induction of tissue inflammation and tail thrombosis in laboratory animals such as rats and mice. It can cause endothelial cell injury and local blood vessel inflammation by releasing inflammatory mediators, which may lead to thrombus formation [29]. Blood vessel inflammation is considered one cause of thrombosis, although a thrombus can lead to inflammation [4]. Circulation disturbances at locations of atherosclerotic plaque rupture stimulate platelet activation and arterial thrombus formation [30]. In this study, we evaluated both extracts in vivo against thrombus formation and found SCC + MAL to be a potent combination compared with SCC or MAL alone; the mixture may be a natural antithrombotic agent with potential to prevent thrombosis or CVD.

SCC + MAL markedly inhibited collagen-induced platelet aggregation through glycoprotein (GP) VI-mediated signaling in platelets. A previous report described MAL (400 *µ*g/mL) inhibition of collagen (2.5 *µ*g/mL)-induced platelet aggregation by 30% [11]. Our results show more potent inhibition of platelet aggregation with SCC + MAL (94 ± 3%) at similar concentrations as 5 *µ*g/mL collagen, indicating that SCC + MAL is an effective natural antiplatelet agent. Cytosolic calcium elevation is mediated through cytosolic release and influx across the plasma membrane [31]. [Ca^2+^]_*i*_ plays a crucial role in platelet activation and increased calcium levels lead to the activation of numerous signaling pathways involved in actin-myosin interaction [32]. We found that SCC + MAL significantly inhibited [Ca^2+^]_*i*_ concentration in collagen-induced platelet aggregation.

Our study demonstrates the inhibitory potential of SCC + MAL on collagen-stimulated platelet aggregation, which is indicated by a clear suppression of [Ca^2+^]_*i*_ mobilization, granule secretion, and integrin *α*_IIb_*β*_3_ activation. Fibrinogen binding to integrin *α*_IIb_*β*_3_ is considered a marker of inside-out signaling; we measured the binding using flow cytometry in the presence or absence of SCC + MAL. Our findings suggest that compromised *α*_IIb_*β*_3_ structural changes may be induced by pretreatment with SCC + MAL. Conversely, these results also imply that outside-in signaling, leading to platelet shape change, may be reduced by SCC + MAL treatment. Platelets are continually exposed to a number of activating factors, such as collagen, thrombin, ADP, fibrinogen, vWF, and inhibitory factors such as prostacyclin (PGI_2_) and ADPase [33]. Thrombosis can be developed if there is disturbance in this equilibrium. Accordingly, for a normal hemostasis, a resilient equilibrium between two routes of platelet activation and inhibition should be critical. Our study proposes that pretreatment of stimulated platelets with SCC + MAL may assist in maintaining this equilibrium.

We observed that SCC + MAL inhibited collagen-stimulated ERK1/2 and JNK but not p38-MAPK activation. A strong inhibitory effect on PI3K/Akt signaling was also observed, showing that both pathways may be involved in modulation of SCC + MAL antiplatelet activity. PI3K inhibitors have been shown to prevent fibrinogen binding to integrin *α*_IIb_*β*_3_ and thrombus formation without affecting coagulation parameters [34]. Previous findings established that PI3K/Akt pathway plays a key role in cardiac protection by inducing antiapoptotic effects and reducing myocardial ischemia-reperfusion injury (MI/RI) [35, 36]. SCC + MAL may potentially exhibit beneficial effects by modulation of PI3K/Akt signaling in reperfusion injury salvage kinase (RISK) pathway. Platelets present MAPK pathway factors such as JNK, ERK and P38 which can be activated by different agonists [24]. Collagen-stimulated ERK2 activation is reliant on TXA_2_ production and ADP secretion [37].

Our findings suggest that SCC + MAL can suppress the GP VI downstream signaling pathway; hence, antagonism of this receptor may characterize a novel therapeutic regimen. Chromatographic separation led to the identification of four compounds in SCC + MAL mixture. Schizandrol A from SCC may be the one that synergistically enhances the antiplatelet effects of MAL. The SCC + MAL mixture inhibited thrombus formation, agonist-stimulated aggregation, [Ca^2+^]_*i*_ mobilization, granule secretion, and *α*_IIb_*β*_3_ activation via MAPK and Akt phosphorylation, demonstrating a potential use of this mixture as an ethnomedicinal antithrombotic agent.

## 5. Conclusion

The SCC + MAL mixture significantly inhibited in vivo thrombus formation and was a potent inhibitor of collagen-induced in vitro platelet aggregation and granule secretion. Our findings indicate that SCC + MAL mixture inhibits agonist-stimulated platelet activation through modulation of downstream signaling via the MAPK and PI3K/Akt pathways.
